# Artificial Intelligence-Driven Skin Parameter Assessment in Indian Adults: Establishing Population-Specific Reference Data for Clinical Dermatology

**DOI:** 10.7759/cureus.109646

**Published:** 2026-05-25

**Authors:** Saurabh Arora, Neha Arora, Manoj Karwa, Surya T Reddy

**Affiliations:** 1 Research, Auriga Research Pvt. Ltd., Delhi, IND; 2 Ningen Skin Sciences, Arbro Pharmaceuticals Pvt. Ltd., Delhi, IND; 3 Clinical Trial and Pharmacovigilance, Auriga Research Pvt. Ltd., Delhi, IND; 4 Clinical Research, Auriga Research Pvt. Ltd., Gurugram, IND

**Keywords:** artificial intelligence, clinical dermatology, dermatology, epidemiology, ethnic skin, population health, reference data, skin assessment

## Abstract

Background

Artificial intelligence (AI)-based skin analysis enables objective, large-scale assessment of dermatologic features; however, population-specific normative reference ranges for Indian skin remain limited. This exploratory study aimed to establish percentile-based reference distributions for facial skin parameters in Indian adults, predominantly comprising Fitzpatrick skin types III-V, who demonstrate distinct pigmentary and aging patterns that are underrepresented in the existing literature.

Methods

This retrospective, cross-sectional study analyzed de-identified facial scan data from 20,914 healthy Indian adults (≥18 years) obtained through the SkinSensAI™ platform (Ningen Skin Sciences Pvt. Ltd., New Delhi, India), of whom 20,913 with complete age data were included in age-stratified analyses. Facial images were processed using an AI engine to generate standardized severity scores (1-5 scale) for 12 facial skin parameters. Descriptive statistics, age-, gender-, and Fitzpatrick-stratified analyses, and inter-parameter correlations were performed in accordance with the Strengthening the Reporting of Observational Studies in Epidemiology (STROBE) guidelines.

Results

Dark circles and enlarged pores emerged as the most prevalent concerns, while wrinkle severity remained low in participants younger than 45 years. Wrinkles showed the strongest correlation with age (r = 0.57, p < 0.001). Male participants exhibited significantly higher scores for pores, spots, wrinkles, and skin laxity than female participants (p < 0.001). Darker Fitzpatrick skin types demonstrated a higher pigmentary burden, whereas wrinkle severity was relatively lower, consistent with melanin-associated photoprotection. Distinct correlation clusters reflected aging-related, pigmentation-sebaceous, and independent skin domains.

Conclusion

This study establishes the first large-scale, AI-derived reference database of facial skin parameters specific to the Indian population. These population-specific benchmarks support objective dermatologic assessment, personalized patient counseling, and equitable AI application in clinical dermatology.

## Introduction

Skin health and appearance are influenced by genetic, environmental, and lifestyle factors, with substantial variation across ethnic populations. India’s diverse population, predominantly comprising Fitzpatrick skin types III-V, exhibits higher melanin content that confers photoprotection against ultraviolet (UV) radiation while also predisposing individuals to pigmentation disorders such as melasma, post-inflammatory hyperpigmentation, and periorbital melanosis [1]. Despite this, large-scale normative data characterizing baseline skin parameters and aging patterns in healthy Indian adults remain limited.

Historically, dermatologic research and clinical grading systems have been derived largely from lighter-skinned populations, resulting in limited applicability to darker phototypes [2]. This creates challenges in both clinical interpretation and AI-based dermatologic assessment, as tools trained on non-diverse datasets may underperform in ethnically diverse populations. The need for culturally representative datasets has been emphasized in recent literature [3].

Advances in artificial intelligence (AI) and computer vision now enable objective, large-scale, and reproducible assessment of multiple skin parameters simultaneously [4]. These technologies offer an opportunity to generate population-level reference datasets that can improve clinical benchmarking and personalized dermatologic care.

Previous studies in Indian populations have examined selected dermatologic features such as pigmentation and aging patterns [5,6], and epidemiological studies have also evaluated the prevalence of sensitive skin [7]. However, these studies were limited in scope and did not utilize AI-driven quantitative approaches.

This study aimed to establish population-specific reference ranges for facial skin parameters in healthy Indian adults using AI-based analysis. Additionally, the study was conducted in accordance with the Strengthening the Reporting of Observational Studies in Epidemiology (STROBE) guidelines [8].

## Materials and methods

Study design and ethical oversight

This was a retrospective, cross-sectional analysis of pre-existing, de-identified facial scan data. The study protocol (ARL/CT/045/25, Version 1.0, dated December 8, 2025) was prospectively approved by an independent Institutional Ethics Committee (approval number GSER-BMR-2025-152, dated December 14, 2025) prior to data analysis. No direct participant contact or experimental intervention occurred. The research adhered to the Declaration of Helsinki [9] and Good Clinical Practice principles throughout. As this was a retrospective analysis of anonymized data, the IEC granted a waiver of prospective informed consent in accordance with Indian Council of Medical Research (ICMR) guidelines [10], recognizing that participants had provided broad consent during initial data collection and that the study posed minimal risk to participants.

Data source

The primary data source was the SkinSensAI platform (Ningen Skin Sciences Pvt. Ltd.), a mobile/web-based application designed to analyze facial photographs and quantify multiple skin parameters. Facial images were automatically processed using the DeepTag AI engine, delivered via Application Programming Interface (API) by BrighTex Bio-Photonics Inc., to extract standardized severity scores for 12 core skin parameters on a 1-5 ordinal scale (1 = minimal/absent, 5 = severe).

The wrinkle parameter, along with other skin parameters, was derived through automated image analysis of standardized facial photographs. Specifically for wrinkle assessment, the algorithm evaluated facial regions for the presence, depth, and distribution of rhytides and fine lines using pre-trained machine learning models trained on large datasets of facial images. Wrinkle severity scores were generated on a standardized 1-5 ordinal scale, with higher values indicating greater severity.

The underlying image processing and feature extraction methods are proprietary to BrighTex Bio-Photonics. However, the output scores are standardized to reflect relative severity in alignment with dermatological grading principles. All images were processed uniformly to ensure consistency and reproducibility across the study population.

The platform additionally assigned Fitzpatrick skin type (I-VI) through algorithmic image analysis and allowed users to self-report skin category (normal, oily, dry, combination, or sensitive). All data retained for analysis were fully de-identified; personally identifiable information, including names, contact information, and precise geographic coordinates, was removed by an independent data manager prior to transfer to the analysis team. Records were then assigned random study identification numbers.

Study population and inclusion/exclusion criteria

This retrospective, cross-sectional study analyzed pre-existing facial scan data from individuals who accessed the SkinSensAI platform between 2023 and 2025. Eligible participants were adults aged ≥18 years who accessed the application from within India, reported good general health without active dermatologic disease, provided informed consent permitting research use of anonymized data, and had complete AI-generated scores for all 12 core facial skin parameters.

Individuals were excluded if they were younger than 18 years, had evidence of acute skin lesions or infections, submitted images of inadequate quality, had incomplete or erroneous scan outputs, or had data that could not be fully de-identified.

After applying these criteria, the final analytical dataset comprised 20,914 unique scans, of which 20,913 had complete age data and were included in age-stratified analyses.

Skin parameters and definitions

Each facial scan was analyzed to generate standardized severity scores for 12 core facial skin parameters using a 1-5 ordinal scale, with higher scores indicating greater severity (Table 1).

**Table 1:** Facial skin parameters and operational definitions Fitzpatrick classification was applied according to Fitzpatrick (1988) [11]. Parameters were derived from the SkinSensAI platform (Ningen Skin Sciences Pvt. Ltd.), powered by the DeepTag™ AI engine developed by BrighTex Bio-Photonics Inc., with reference to publicly available documentation [12,13]. Use of the platform was conducted with the agreement of Ningen Skin Sciences Pvt. Ltd. under its commercial association with BrighTex Bio-Photonics Inc. Definitions were developed by the authors specifically for this study and were not adapted from prior sources; therefore, no copyright permission was required.

Skin parameter	Operational definition
Pores	Visibility and prominence of facial pores
Spots	Hyperpigmented lesions, including ultraviolet-related lentigines and melasma-like marks
Dehydration	Indicators of surface dehydration, such as fine dehydration lines
Texture	Degree of skin surface irregularity and roughness
Wrinkles	Presence and severity of facial lines, rhytides, and wrinkle depth
Dark circles	Periorbital hyperpigmentation or under-eye darkness
Oiliness	Level of sebaceous activity and facial oil production
Redness	Facial erythema involving areas such as the cheeks, nose, or forehead
Uneven skin tone	Blotchy or non-uniform pigmentation distinct from discrete pigmented spots
Acne	Presence and severity of acne lesions or inflammatory papules
Elasticity	Reduction in skin elasticity or tendency toward sagging
Firmness	Decreased skin firmness or visible laxity

Fitzpatrick skin type (types I-VI) was determined algorithmically, with type I representing very fair skin and type VI representing deeply pigmented skin, based on the classification originally described by Fitzpatrick [11]. Self-reported skin category (normal, oily, dry, combination, or sensitive) was collected based on participant perception and was available for approximately 28% of the study population.

Statistical analysis

All statistical analyses were conducted in accordance with a pre-specified analysis plan and are reported in accordance with the STROBE guidelines [8]. Descriptive statistics were used to summarize participant characteristics and skin parameter distributions, including means, standard deviations, medians, interquartile ranges, and selected percentiles (5th, 25th, 50th, 75th, and 95th). Percentile ranges (5th-95th) were used to establish population reference benchmarks.

Stratified analyses were performed across key demographic variables, including age (categorized into 18-25, 26-35, 36-45, 46-55, and ≥56 years), gender (male vs. female), and Fitzpatrick skin type (types III-VI, with types I-II excluded from detailed comparison because of the small sample size). An exploratory subset analysis was additionally conducted based on self-reported skin category.

Normality of continuous variables was assessed using the Shapiro-Wilk test. Between-group comparisons were performed using the Student’s t-test or Mann-Whitney U test, as appropriate. Multi-group comparisons employed one-way analysis of variance (ANOVA) or the Kruskal-Wallis test, with post hoc testing using Tukey’s or Dunn’s method where applicable. Statistical significance was defined as a two-sided p-value < 0.05, with Bonferroni correction applied for multiple comparisons in subgroup analyses.

Associations between skin parameters and age were assessed using Pearson or Spearman correlation coefficients, depending on data distribution. Correlation strength was interpreted as strong (|r| > 0.5), moderate (0.3-0.5), or weak (0.1-0.3). A full pairwise correlation matrix was generated and visualized using a heatmap to identify co-occurring skin phenotypes.

All analyses were performed using Python (Python Software Foundation), including the Pandas and SciPy libraries, with key descriptive outputs cross-validated using IBM SPSS Statistics for Windows.

Methodological considerations

The proprietary DeepTag algorithm lacks full transparency, and the AI-derived scores were not calibrated against dermatologist clinical ratings, necessitating external validation despite biologically consistent trends, such as age-related wrinkle progression and the correlation between pore size and oiliness. Dehydration and texture demonstrated perfect correlation (r = 1.00), indicating possible algorithmic redundancy and reducing the effective number of independent parameters to 11. All AI-generated skin scores are quantitative, non-diagnostic measures intended for population-level research only and should not replace formal dermatologic evaluation.

## Results

The demographic characteristics of the study population are summarized in Table 2. A total of 20,914 participants were included, of whom 15,616 (74.7%) were female. The mean age was 32.1 ± 8.45 years (range: 18-69 years), with a median age of 30 years (interquartile range (IQR): 26-36). The cohort was predominantly young, with 4,508 (21.6%) aged 18-25 years, 9,884 (47.3%) aged 26-35 years, and 5,126 (24.5%) aged 36-45 years. Only 5.8% (1,207) were older than 45 years, and fewer than 1% were aged ≥56 years, reflecting lower platform adoption among older age groups.

**Table 2:** Demographic characteristics of study population (N = 20,914) Participant demographics included age, gender, Fitzpatrick skin type, and self-reported skin category. Values are presented as number (percentage), unless otherwise indicated. Age is reported as mean ± standard deviation. Fitzpatrick skin types IV and V were the most prevalent in the study population. Note: Fitzpatrick skin type was successfully classified in 20,911 (99.99%) participants; 3 (0.01%) were excluded because of insufficient algorithm confidence, without affecting the overall analysis.

Characteristic	N	% of total
Total participants	20,914	100
Age (years)	32.1	8.45
18-25	4,508	21.6
26-35	9,884	47.3
36-45	5,126	24.5
46-55	1,207	5.8
≥56	189	0.9
Gender		
Female	15,616	74.7
Male	5,298	25.3
Fitzpatrick skin type		
Type I (very fair)	34	0.16
Type II (fair)	99	0.5
Type III (fair beige)	2,161	10.3
Type IV (olive/light brown)	8,771	42
Type V (brown)	8,812	42.1
Type VI (dark brown)	1,034	4.9

Fitzpatrick skin types IV (8,771, 42.0%) and V (8,812, 42.1%) predominated, followed by type III (2,161, 10.3%) and type VI (1,034, 4.9%). Types I (34, 0.16%) and II (99, 0.5%) together accounted for <1% of participants, consistent with the expected epidemiology of the Indian population (Table 2).

Overall distribution of skin parameters

The distribution of all facial skin parameters is presented in Table 3. Among the evaluated features, dark circles and enlarged pores emerged as the most prevalent concerns.

**Table 3:** Distribution of facial skin parameter severity scores in the study population (N = 20,911) All parameters were scored on a standardized 1-5 ordinal scale, with higher values indicating greater severity. Dehydration and texture showed perfect correlation (r = 1.0), indicating algorithmic redundancy. IQR = interquartile range (25th-75th percentile). Note: Fitzpatrick skin type was successfully classified in 20,911 (99.99%) participants; 3 (0.01%) were excluded because of insufficient algorithm confidence, without affecting the overall analysis.

Parameter	Mean Score	SD	Median (IQR)	5th-95th percentile	Min-max	Clinical interpretation
Pores	3.56	1.3	4 (3-5)	1-5	1-5	Visible pore prominence; higher = enlarged pores
Spots	2.02	0.94	2 (1-3)	1-4	1-5	Hyperpigmented lesions/UV spots; higher = more spots
Dehydration	2.99	0.92	3 (2-4)	1-4	1-5	Surface dehydration/fine lines; note: r = 1.0 with texture
Texture	2.99	0.92	3 (2-4)	1-4	1-5	Roughness/irregularity; redundant with dehydration
Wrinkles	2.15	0.64	2 (2-2)	1-3	1-5	Rhytides depth; higher = more prominent lines
Dark circles	3.63	0.88	4 (3-4)	2-5	1-5	Periorbital hyperpigmentation; ubiquitous in cohort
Oiliness	2.99	0.78	3 (2-4)	2-4	1-5	Sebaceous activity/shine; higher = more oily
Redness	1.38	0.5	1 (1-2)	1-2	1-4	Facial erythema; minimal in healthy cohort
Uneven tone	1.71	0.56	2 (1-2)	1-2	1-5	Blotchiness distinct from discrete spots
Acne	1.32	0.53	1 (1-2)	1-2	1-5	Acne lesions; minimal in healthy adults
Elasticity	3.64	1.1	4 (3-5)	2-5	1-5	Loss of elasticity/sagging tendency
Firmness	3.75	1.08	4 (3-5)	2-5	1-5	Skin laxity/reduced turgidity

Dark circles demonstrated a mean score of 3.63 ± 0.88 and a median of 4 (IQR: 3-4), with the 5th-95th percentile range spanning 2-5, indicating that the vast majority of participants exhibited at least mild periorbital pigmentation. Enlarged pores showed a mean score of 3.56 ± 1.30 and a median of 4 (IQR: 3-5), with more than 75% of participants scoring ≥3, reflecting widespread pore visibility.

Despite the relatively young age of the cohort, elasticity (mean 3.64 ± 1.10) and firmness (mean 3.75 ± 1.08) scores were moderately elevated, with more than half of participants scoring ≥4. These parameters demonstrated strong inter-correlation (r ≈ 0.87).

In contrast, wrinkle severity was low overall, with a mean score of 2.15 ± 0.64, and >95% of participants scored ≤3. Acne and redness were minimal across the cohort (mean scores 1.32 and 1.38, respectively), consistent with a healthy, non-diseased population.

Reference percentile ranges derived from Table 3 enable clinical benchmarking. For example, wrinkles showed a 5th-95th percentile range of 1-3, while acne ranged from 1-2, indicating that higher scores represent outliers within this population.

Self-reported skin category data were available for 5,942 participants (28.4%), among whom oily skin was most common (48.5%), followed by combination (27.0%), normal (20.9%), and dry skin (3.6%). No participants selected “sensitive.”

Participants represented 32 of India's 36 states and union territories, with the highest representation from West Bengal (5,439; 26.0%), followed by Odisha (2,240; 10.7%), Maharashtra (1,776; 8.5%), Assam (987; 4.7%), and Delhi NCT (978; 4.7%), indicating broad geographic coverage across the Indian subcontinent (Table 4). A small proportion of participants were of international origin (n=22; 0.1%), with the vast majority being Indian (n=20,892; 99.9%).

**Table 4:** Geographic distribution of participants Note: Dataset included participants from 32 of India's 36 states and union territories, with negligible international representation (n=22, 0.1%).

State / Union Territory	Participants	Percentage (%)
West Bengal	5,439	26.0
Odisha	2,240	10.7
Maharashtra	1,776	8.5
Assam	987	4.7
Delhi NCT	978	4.7
Uttar Pradesh	871	4.2
Punjab	671	3.2
Haryana	346	1.7
Others (24 states/UTs)	8,584	41.1
International	22	0.1
Total Indian	20,892	99.9

Age-stratified analysis

**Figure 1:**
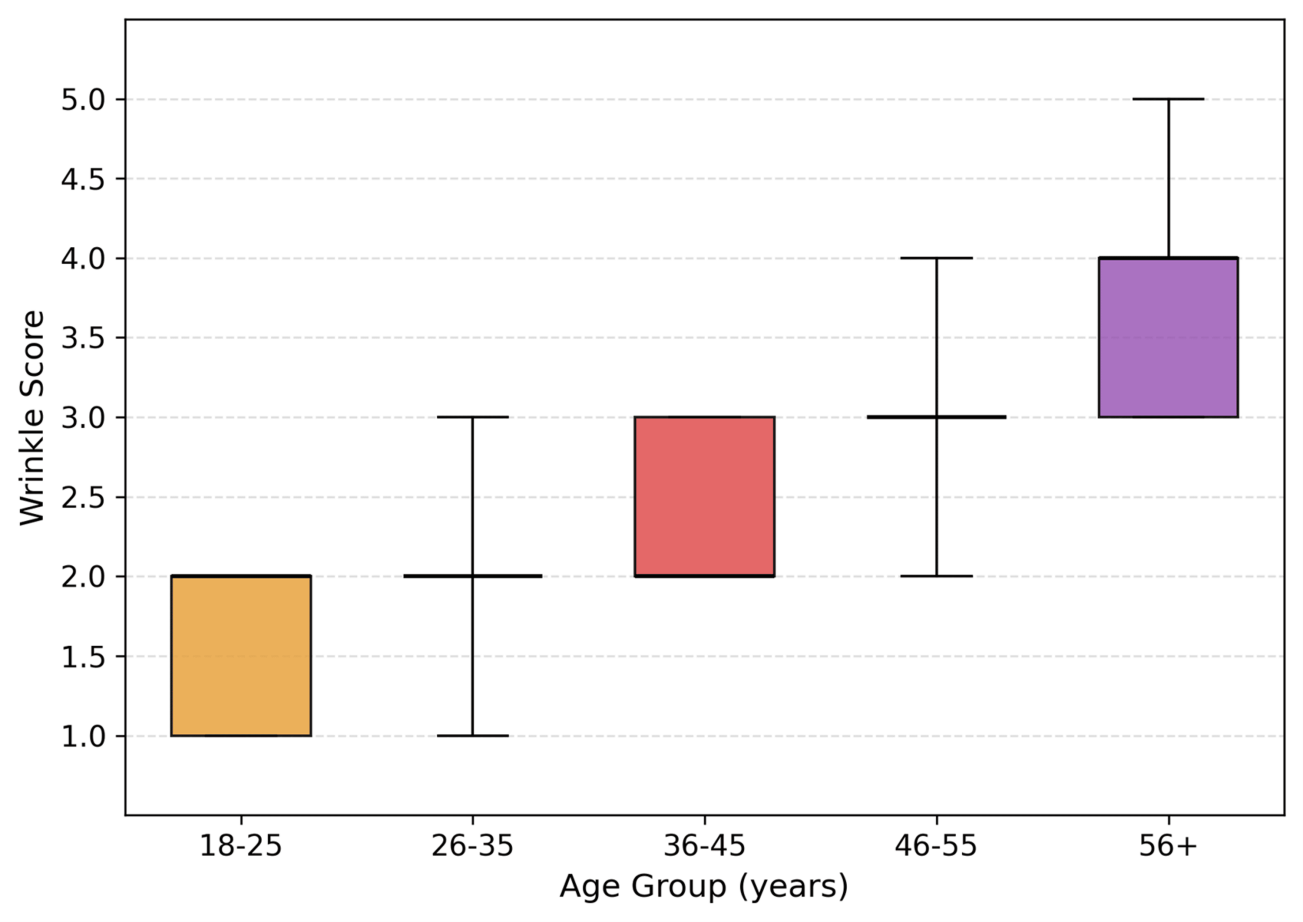
Facial wrinkle severity stratified by age group Boxplots illustrate the distribution of wrinkle severity scores across age groups. The median is represented by the central line, and the interquartile range (IQR) by the box. Whiskers extend to the 5th-95th percentiles, with outliers omitted for clarity. A progressive increase in wrinkle severity with age is observed, with median scores rising from approximately 1 in the 18-25-year group to 4 in individuals aged ≥56 years, accompanied by increasing variability in older age groups.

Age demonstrated a strong association with multiple skin parameters, most notably wrinkles. As shown in Figure 1, median wrinkle scores increased progressively from 1 in the 18-25-year group to 2 in 26-45 years, 3 in 46-55 years, and 4 in ≥56 years. Mean wrinkle scores increased approximately linearly across decades (p < 0.001), with a Pearson correlation coefficient of r = 0.57, representing the strongest age-related association observed. 

Elasticity and firmness similarly increased with age (r = 0.49 and r = 0.46, respectively; both p < 0.0001), indicating progressive structural laxity. Hyperpigmented spots showed a moderate positive correlation with age (r = 0.30, p < 0.001), with the prevalence of moderate-to-severe spots (score ≥3) increasing from approximately 15% in participants aged 18-25 years to >50% in those older than 45 years.

Dark circles showed only a weak correlation with age (r = 0.18), with consistently high mean scores across all age groups. Acne and oiliness demonstrated weak negative correlations with age (both r ≈ -0.12), reflecting higher prevalence in younger adults and a gradual decline with advancing age.

Gender-stratified analysis

Significant gender-based differences were observed across multiple parameters. Male participants exhibited higher mean scores for pores, spots, wrinkles, elasticity, firmness, and dehydration/texture compared with females (all p < 0.001). For example, mean pore scores were 4.05 in males versus 3.40 in females, and mean wrinkle scores were 2.40 versus 2.07, respectively (Table 5).

**Table 5:** Gender-stratified analysis Mean severity scores of selected facial skin parameters stratified by gender. Values represent mean scores on a standardized 1-5 ordinal scale, with higher values indicating greater severity. Statistical significance was assessed using appropriate comparative tests, with p < 0.05 considered significant.

Parameter	Male	Female	p-value
Pores	4.05	3.4	<0.001
Wrinkles	2.4	2.07	<0.001
Oiliness	2.88	3.03	<0.001
Acne	1.21	1.36	<0.001

Females showed modestly higher scores for oiliness (3.03 vs. 2.88) and acne (1.36 vs. 1.21) (both p < 0.001). Redness and uneven skin tone did not differ meaningfully by gender. These differences persisted for several parameters even after accounting for age differences between genders.

Fitzpatrick skin type analysis

Comparisons across Fitzpatrick skin types III-VI revealed phototype-dependent patterns. Darker skin types (V-VI) demonstrated significantly higher pigmentation-related scores, including spots and dark circles, compared with type III (p < 0.001). Uneven skin tone was also marginally higher in darker phototypes, although absolute differences were small (Table 6).

**Table 6:** Fitzpatrick skin type analysis Comparison of mean severity scores of selected facial skin parameters across Fitzpatrick skin types. Values represent mean scores on a standardized 1-5 ordinal scale, with higher values indicating greater severity. Statistical significance was assessed using appropriate comparative tests, with p < 0.05 considered significant.

Parameter	Type III	Type IV-VI	p-value
Spots	Lower	Higher	<0.001
Dark circles	Lower	Higher	<0.001

Wrinkle severity tended to be slightly higher in lighter skin types, consistent with melanin-associated photoprotection, although age differences between groups may partially confound this finding. Pore size and oiliness did not show a consistent gradient across skin types, with all phototypes exhibiting relatively high average pore scores.

Inter-parameter correlations and phenotypic clusters

**Figure 2:**
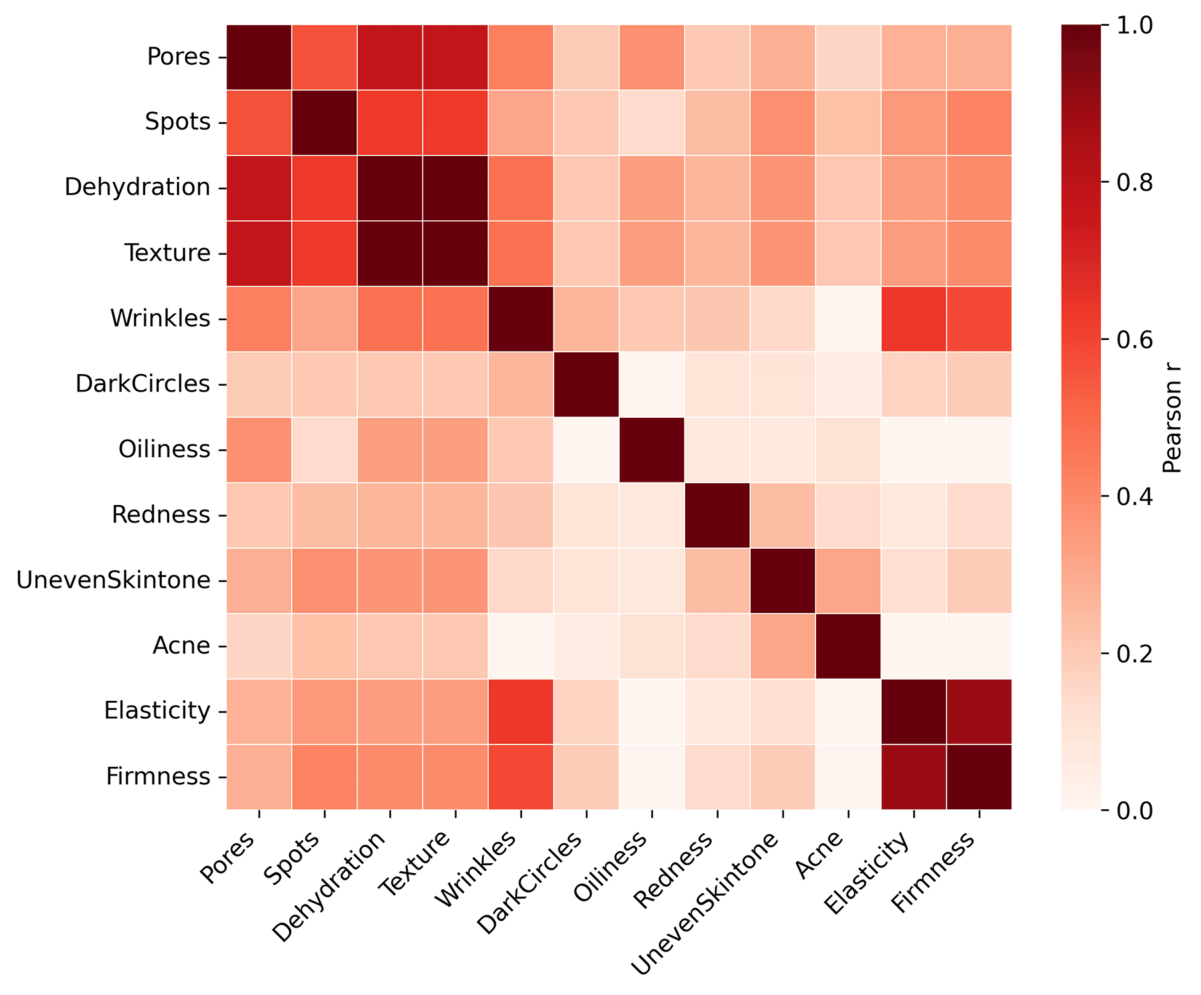
Correlation between facial skin parameters Heatmap illustrating pairwise Pearson correlation coefficients between facial skin parameters. Warmer colors indicate stronger positive correlations, while cooler tones represent weaker or negative associations. Strong correlations are observed between elasticity and firmness, as well as between wrinkles and structural parameters, while features such as acne and dark circles show relatively weaker associations with other variables. All parameters were derived using AI-based facial image analysis on a standardized 1-5 ordinal severity scale.

Pairwise correlations among skin parameters are illustrated in Figure 2. Strong correlations were observed between elasticity and firmness (r ≈ 0.87), as well as between wrinkles and elasticity or firmness (r ≈ 0.62-0.66). Pores and texture were also strongly correlated (r ≈ 0.78). Dehydration and texture demonstrated perfect correlation (r = 1.00), indicating algorithmic redundancy.

Moderate correlations were noted between spots and uneven tone (r ≈ 0.40) and between pores and spots (r ≈ 0.59). Dark circles and acne showed weak correlations with most other parameters, suggesting relative independence.

These patterns defined four phenotypic domains: aging/structural, pigmentation, sebaceous/texture, and an independent domain dominated by dark circles.

Co-prevalence of high-severity parameters

Approximately 30% of participants exhibited at least one skin parameter with a severity score ≥4, while only 5% had three or more high-severity parameters, as shown in Table 7. The most frequent co-occurrence was dark circles with enlarged pores, reflecting their high prevalence. Fewer than 1% of participants exhibited high severity across nearly all parameters, representing potential outliers with disproportionate skin damage relative to age.

**Table 7:** Co-prevalence of high-severity parameters Distribution of participants based on the number of facial skin parameters with high severity (score ≥ 4). Values represent the percentage of participants within the study population.

Category	Percentage of participants (%)
At least one parameter with a severity score ≥4	30
Three or more parameters with a severity score ≥4	5
High severity across most parameters (≥8 parameters)	<1

## Discussion

This analysis provides the first comprehensive, population-specific reference dataset of facial skin parameters in healthy Indian adults. The findings quantitatively confirm long-standing clinical observations that Indian skin aging is characterized predominantly by pigmentary and textural concerns rather than early wrinkle formation, while also establishing objective benchmarks for clinical interpretation.

**Figure 3:**
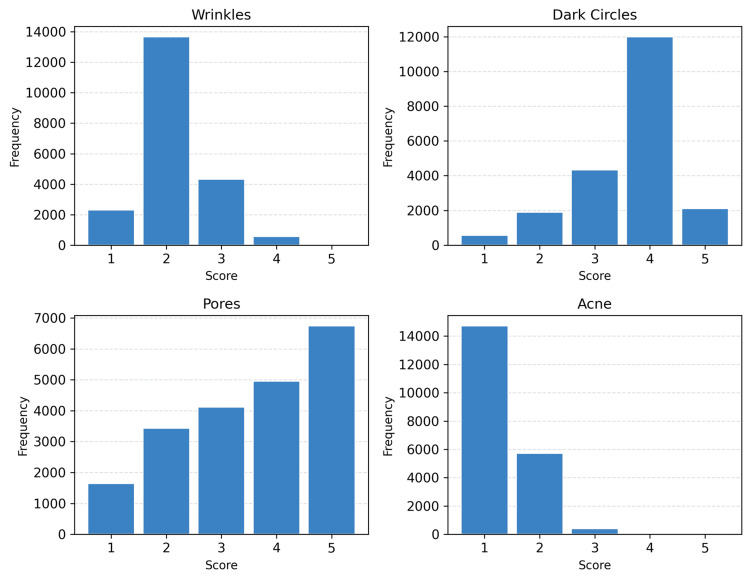
Distribution of selected facial skin parameter scores in the study population Wrinkles (facial lines): The histogram is sharply peaked at score 2, indicating that the vast majority of individuals show only minimal fine lines; very few reach scores 4-5 (severe wrinkles) in this cohort. Dark circles (under-eye pigmentation): Scores are skewed toward higher values, with many participants demonstrating moderate-to-high dark circle severity (mode at 4). Pores (enlarged pores): A substantial proportion of participants exhibit high pore visibility (scores 4-5), making this a common concern. Acne: Most participants have minimal acne (score 1), with frequencies declining steeply at higher scores, reflecting the relatively low prevalence of active acne breakouts in this cohort. In each plot, the y-axis represents the number of individuals (out of approximately 20,000) corresponding to each severity score. Note: This figure is original and was generated as part of the present study using AI-derived data.

Pigmentary features, particularly periorbital dark circles and hyperpigmented spots, were the most prevalent concerns across all age groups [5,6,14]. Dark circles demonstrated high median severity even in young adults and showed only a weak correlation with age, suggesting a largely constitutional or genetic basis rather than a purely age-related phenomenon. In contrast, wrinkle severity remained low in most participants under 45 years, with more than 75% scoring ≤2, consistent with the proposed photoprotective role of higher melanin content in Indian skin. The observed increase in wrinkle severity after midlife suggests an age-associated transition toward greater structural aging features. Skin aging patterns in Indian populations differ from those of Caucasian cohorts, with delayed wrinkle onset [6,15]. In summary, the overall distribution analysis indicates that pigmentation-related issues (dark circles, spots) and texture/sebum-related issues (pores, oiliness) are prevalent in this large Indian cohort, whereas classical signs of aging (wrinkles, deep sagging) are not yet prominent in the majority, likely reflecting the relatively young age distribution of the sample. Figure 3 visualizes these patterns, with dark circles and pores (blue histograms) shifting toward higher scores compared with wrinkles and acne, which cluster at lower severity levels.

Gender-based analyses revealed higher severity of pores, pigmentation, wrinkles, and structural laxity in men, likely reflecting lower sunscreen use and greater cumulative ultraviolet exposure [16,17], identifying Indian men as an underserved group in preventive dermatology [18]. Fitzpatrick skin type-specific patterns further demonstrated that darker phototypes carry a higher pigmentary burden, while lighter phototypes showed marginally greater wrinkle severity, consistent with the recognized association between melanin biology, photoprotection, and pigmentation-related variation [1,19]. These findings should be interpreted cautiously, as demographic differences between subgroups may partially contribute to the observed associations.

Inter-parameter correlation analysis identified distinct phenotypic domains, including an aging/structural cluster (wrinkles, elasticity, firmness), a pigmentation-sebaceous cluster (spots, pores, oiliness, texture), and an independent dark-circle domain. These patterns support combination, phenotype-driven therapeutic strategies rather than isolated parameter treatment [20].

Clinically, the reference percentile ranges generated in this study enable objective benchmarking and personalized counseling. Although limitations include selection bias toward younger, urban users and a lack of external clinical validation, the large sample size and biological plausibility support the robustness of relative comparisons. Advances in AI-based dermatologic assessment have enabled objective and scalable skin evaluation [4,14,21], supporting improved clinical decision-making. Overall, this work demonstrates the value of AI-enabled, population-specific dermatologic data in advancing precision and equitable dermatology [22].

This study has several limitations. First, the dataset is derived from a mobile application and may be biased toward younger, urban, and technology-literate users. Second, AI-derived scores were not externally validated against dermatologists' clinical assessments. Third, the proprietary nature of the algorithm limits transparency and reproducibility. Finally, the cross-sectional, retrospective design precludes causal inference and limits longitudinal assessment of temporal skin changes over time.

## Conclusions

This study establishes population-specific reference ranges for facial skin parameters in Indian adults using AI-based analysis. The findings highlight the predominance of pigmentary and textural concerns, particularly periorbital dark circles, enlarged pores, and oiliness, over wrinkle-based aging, in contrast to patterns typically observed in lighter-skinned populations. Wrinkle severity and structural laxity remained relatively mild in individuals under 50 years, supporting the photoprotective role of higher melanin content.

Significant variation was observed across age, gender, and Fitzpatrick skin type, with men demonstrating greater photodamage and darker phototypes showing a higher pigmentary burden. Inter-parameter correlations revealed distinct biological domains, supporting phenotype-based, combination therapeutic approaches. These population-specific benchmarks enable objective clinical assessment and personalized counseling, while also highlighting the value of AI-driven, population-level dermatologic research. Future studies incorporating longitudinal follow-up and external validation will further strengthen these findings and advance precision dermatology for Indian skin.
