# Correction: Artificial Intelligence-Driven Skin Parameter Assessment in Indian Adults: Establishing Population-Specific Reference Data for Clinical Dermatology

**DOI:** 10.7759/cureus.c440

**Published:** 2026-05-27

**Authors:** Saurabh Arora, Neha Arora, Manoj Karwa, Surya T Reddy

**Affiliations:** 1 Research, Auriga Research Pvt. Ltd., Delhi, IND; 2 Ningen Skin Sciences, Arbro Pharmaceuticals Pvt. Ltd., Delhi, IND; 3 Clinical Trial and Pharmacovigilance, Auriga Research Pvt. Ltd., Delhi, IND; 4 Clinical Research, Auriga Research Pvt. Ltd., Gurugram, IND

This article has been corrected to revise Table 4 and the preceding paragraph to fix incorrect state-wide distribution data. This change does not affect the study's conclusions.

